# The effect of multimedia education on patients’ health knowledge: A systematic review and narrative synthesis

**DOI:** 10.12968/bjcn.2025.0105

**Published:** 2026-01-02

**Authors:** Kathryn Jack, Ultan Allen, Philippa Matthews, Ahmed Elsharkawy, Stephen Ryder, Elizabeth Hendron, Sarah Williams

**Affiliations:** 1 National Institute for Health Research Nottingham Biomedical Research Centre, University of Nottingham and Nottingham University Hospitals NHS Trust, Nottingham, UK; The Nottingham Centre for Evidence-Based Healthcare (CEBHC), University of Nottingham, Nottingham, UK; 2 Nottingham University Hospitals NHS Trust, Nottingham, UK; 3 The Francis Crick Institute, 1 Midland Road, London, UK; Division of Infection and Immunity, University College London, Gower Street, London, UK; Department of Infectious Diseases, University College London Hospital, Euston Road, London, UK; 4 Liver Unit and NIHR Biomedical Research Centre at University Hospitals Birmingham NHS Trust and University of Birmingham, Edgbaston, Birmingham, UK; 5 National Institute for Health Research Nottingham Biomedical Research Centre, University of Nottingham and Nottingham University Hospitals NHS Trust, Nottingham, UK; 6 Nottingham University Hospitals NHS Trust, Nottingham, UK; 7 British Liver Trust, Venta Court, 20 Jewry Street, Winchester, UK

## Introduction

The delivery of health-related information across the full spectrum of activities necessary to either prevent or optimize the outcomes of an acute healthcare event, support a short-term intervention or episode, or facilitate the optimal management of a long-term condition (LTC) in a hospital or community setting, is a fundamental component of all healthcare professionals’ roles. Contemporary healthcare delivery is underpinned by the tenet that individuals are fully informed and empowered to make informed decisions, with the phrase “no decision about me, without me” directing this approach [1] (p.3). Meeting the goals of improving health-related outcomes and reducing health inequalities requires the closure of gaps in health knowledge, clarifying misunderstandings, and correcting misinformation through the timely delivery of evidence-based health information and advice [2,3]. However, written health education materials do not always meet individuals’ reading ability [4] so there is a need to consider alternative approaches.

Multimedia formats, defined as written or spoken words plus static or dynamic pictures, support a dual-processing theory that people have separate cognitive channels for verbal and visual information through which to process information, each of which has their own limited capacity [5] (p.34). This cognitive theory of multimedia learning (CTML) asserts that meaningful learning is an active process whereby visual and auditory materials are combined and integrated into their long-term knowledge [6]. Combining visual and auditory or textual materials can support understanding and information recall amongst people who have lower levels of health literacy [7,8]. Therefore, the following systematic re-view question was constructed to ascertain if multimedia education tools (MmET) could increase patients’ knowledge and understanding of health information: What is the effectiveness of multimedia education tools versus standard of care or written educational materials on patients’ knowledge and understanding of health information?

In this paper, we use the term “patient” when referring to the individuals who are receiving the multimedia health education described. However, we recognise that this word has been described by some as being both paternalistic and unsuitable for people who live with a chronic health condition or are in a community environment [9]. The UK National Institute for Health and Care Research guidance on working with “People and Communities” explain these groups could include patients, service users, carers, and members of the public [10]. For simplicity in this paper, the term “patient” refers to all people receiving education about their health issue from a healthcare professional. The authors’ rationale for undertaking this review stems from a need to explore ways in which to improve the access to and content of information about hepatitis B virus (HBV) infection amongst global majority populations from underserved communities whose health literacy is poor. Once people engage with specialist viral hepatitis healthcare professionals in a hospital, access to education is readily available. However, HBV testing is usually undertaken in a community healthcare environment and thus pre-hospital education at the point of diagnosis is important to improve the linkage to care pathways.

## Materials and Methods

### Protocol and registration

The protocol for this systematic review is registered at: PROSPERO (International Prospective Register of Systematic Reviews); ID CRD 42024518647; March 2024. The systematic review of randomised controlled trials presented in this paper was undertaken to securely confirm the effectiveness of MmET in contrast to a control group. This review follows the Joanna Briggs Institute (JBI) methodological guidance [11] and is reported in accordance with the PRISMA-P (Preferred Re-porting Items for Systematic Reviews and Meta-Analyses) checklist [12].

### Inclusion and exclusion criteria

We included all randomized controlled and quasi-experimental studies reported between January 2014 and February 2024 that were written in English including: (i) the comparison of a MmET intervention to the current standard of care (a passive comparator); (ii) the comparison to an alternative additional intervention such as written material or verbal information (an active comparator); (iii) with one or more intervention arms; (iv) adults aged 18 years and over; (v) all participant characteristics including gender, ethnicity, clinical condition or diagnosis, or the professional qualification or status of the healthcare professional delivering the intervention; (vi) all healthcare locations either in the community or hospital; and (vii) studies that reported an objective quantitative pre and post level of knowledge and understanding of the health information delivered following the intervention. The inclusion criteria timeframe was chosen to ensure that the social context was contemporary.

We excluded studies that used multimedia tools for teaching healthcare professionals, for gaining consent for research participation, or for use by pharmaceutical companies for any direct to customer drug advertising.

### Literature search and study selection

A three-step literature search strategy was used for this review which was conducted on 23rd February 2024. For the purposes of this review, we defined multimedia (MM) as either a video with people, or an animation, combined with spoken words. The MmET may be hosted on the internet, a locally held intranet, or on an individual electronic device. Participants may be able to access the MmET using a handheld device such as a smartphone or tablet, or a desktop personal or laptop computer. The purpose of the MmET will have been to deliver health information and educational material to adult patients to increase their knowledge about their diagnosis, investigations or procedures, their treatment, and disease management including prevention of further deterioration approaches. Firstly, to construct the search strategy we conducted, with a professional librarian (EH) who specializes in literature searching, an initial brief scan of the MEDLINE, Embase, and Emcare databases to identify publications of relevance and written in English. Secondly, we identified the key words and index terms in the titles and abstracts of relevant articles to develop a full search strategy. The search was conducted (via Ovid) on 23 February 2024 of MEDLINE, EMBASE and EM-CARE (see Box I). Finally, the reference lists of all the included studies were screened for additional studies. The search results are presented in the PRISMA flow diagram (figure I) [12]. The search records were imported into a reference management system (JBI SUMMARI), duplicates removed and then screened according to their title and abstract. The abstracts initially considered for inclusion were screened independently by two authors, and those deemed appropriate for a full text review were assessed independently by three authors. Disagreements were resolved through discussion to reach a consensus.

### Data extraction

Data on the study characteristics and outcomes (e.g. country, number of participants, clinical condition or disease, nature of the intervention, method of assessing the impact of the intervention, and quantitative outcomes) were extracted by the lead author (KJ), discussed with a second author (UA), and recorded in an excel spreadsheet. These data detailing the effects of the MmETs were synthesized into thematic areas and narratively reported.

### Quality assessment

The publications selected for final inclusion were assessed for their methodological quality using the JBI critical appraisal tool for randomised controlled trials by two authors, one who performed the assessment and a second who verified the assessment [9]. Disagreements were resolved and a consensus reached through discussions.

### Data synthesis

The narrative synthesis approach to presenting the results is a pragmatic solution to the heterogeneity of methods for data collection and reporting outcomes within the included studies that pre-vents a meta-analysis of data.

## Results

### Overview of study characteristics

The literature search identified 15 publications for inclusion (Figure I), published between 2014 and 2021. The studies were conducted in the US (n=6) [13–18], Canada (n=3) [19–21], China (n=2) [22,23], and the UK, Australia, Netherlands, and Singapore (n=1 per country) [24–27]. The MmET were constructed to aid the delivery of health information about a procedure or intervention (n=10) [13–15,17,18,22,23,25–27] and long term-condition management (n=5) [16,19–21,24]. An overview of the study characteristics is shown in Table I. The JBI critical appraisal tool for randomised controlled trials (RCTs) comprises 13 questions that assess the risk of bias, or systemic error, in three domains: the study design (questions 1-6,13); outcomes (questions 7-12); and results (questions 10-12) [14]. Table II shows the quality scores of the studies according to the JBI appraisal tool. There was no risk of bias for all 15 studies in the domains covered by questions 3, and 8-13. However, in this review we included both randomised and quasi-experimental studies. Therefore, 2/15 publications reported pilot studies [13,14] and 5/15 did not include a formal sample size calculation [13,19,21,23,26]. Furthermore, two studies that did have a sample size calculation did not recruit to target [16,20], so overall 7/15 of the publications reviewed were underpowered and may have underestimated the true extent of their intervention’s effect.

### Narrative summary

A narrative analysis and summary of the 15 studies was conducted by the lead author, and checked and discussed by a second author (UA), to identify and synthesize data and study details relevant to our research question: What is the effectiveness of multimedia education tools versus standard of care or written educational materials on adults’ knowledge and understanding of health information? In this section, we report on three synthesised findings drawn from the data and information in the studies.

### MmET effectiveness

Out of the 15 studies, 11 showed that a multimedia approach to information delivery can lead to an increase in knowledge and understanding. Of the 15 studies, ten provided pre-procedural information and five addressed the management of long-term conditions. Of the four studies where there was no clear pre-post intervention benefit, two did not have a sample size calculation [19,21] and one study did not recruit to target [16], so there is the possibility of a type II statistical error contributing to their results. Among the pre-procedural studies, nine out of ten showed an increase in participants’ knowledge [13,15,17,18,22,23,25–27], while two out of five studies on long term management also reported similar improvements [20,24]. Five studies measured knowledge retention at between 2 weeks and 6 months following the intervention delivery [17,20,21,24,25], of which three demonstrated a degree of prolonged knowledge retention [17,20,24]. None of the studies included in this review incorporated any assessments of long-term engagement in clinical services or health outcomes.

### MmET Components and characteristics

The most frequently used multimedia style was an animation [13–15,17,19,21,25–27], with three studies combining the visual material with spoken words [13,14,27], four studies including spoken voice and written text [15,17,25,26], and two studies with written text but no voice [19,21]. A further four studies used videos of people speaking [18,20,22,23], and the two remaining studies included both animated and video visual material, with both written and spoken accompaniments [16,24]. The majority of publications did not provide links to the interventions so that readers could view the interventions. Four papers included pictures of screenshots of a limited example of the intervention content [16,25–27]. Of the seven papers that included details of how to access the online MmET material, three did not work [18,19,21], one contained only short extracts [24], one was narrated in Dutch with no other language subtitles [26], leaving only two MmET interventions that could be viewed [14,27]. Written details of the interventions were however reported in the publications. The interventions’ running time were variable and ranged between three and 40 minutes for a single video or animation, with studies detailing a series of five x 5-minute animations [21] and seven videos totalling 60 minutes [24]. Six of the studies included interactive components to aid learning, such as a quiz or labelling a diagram [13,15–17,21,24]. Five of the six videos (with people) and six of the nine animation MmET interventions led to an increase in participants’ knowledge. Three studies [14,23,26] referred to the CTML, which is underpinned by 15 evidence-based features [6]. However, there was insufficient detail reported in the three studies with which to assess full fidelity to these principles. Overall, there was a high degree of variation in the MmET components between studies.

### Health Literacy and language

Health literacy was assessed in only three of the 15 studies [14,15,24], all of whom reported low levels amongst their study populations. The majority of the studies were conducted in high-income countries, with just two from an upper middle-income country, namely China (see Table I). One study from Canada [20] focused on a Chinese patient population who although spoke Canadian- English as a second language, required a culturally and linguistically specific education. Their video with spoken voice intervention led to an increase in participants’ knowledge and understanding of their chronic health condition and rehabilitation process (p< 0.05), and in the technical accuracy of their medical device use (p< 0.001). All other studies delivered their interventions to a population in their native language. A study conducted in the Netherlands sought to determine the effect of different multimedia formats amongst people with varying health literacy [26]. The results showed that spoken messages were recalled more accurately than written (p= 0.02), and that participants reacted more positively to the information (p= 0.01). Furthermore, amongst people with a lower health literacy, information delivered in an animation format with spoken text resulted in higher recall scores and an increased positive reaction (p= 0.04) [26].

## 4 Discussion

This review sought evidence to understand the effectiveness of multimedia education tools versus standard of care or written educational materials on patients’ knowledge and understanding of health information. Overall, the majority of studies signal that MmET interventions can lead to an increase in individuals’ health related knowledge. Health education interventions that meet the needs of the intended population will underpin all three functions of public health practice, these being health protection, health improvement, and health service delivery. These domains all first require that people who live with or are at risk of whichever disease is being discussed, can comprehend and retain the healthcare professionals’ messages designed to optimize their individual and collective morbidity and mortality rates. Individuals’ health literacy levels, defined as the extent to which people can access, interpret and understand the basic health information and services they need to make decisions regarding their health and difficulties [28] are a key determinant of success in any health education programme. However, only three studies [16,17,26] included in this review acknowledged this was a rate determining factor of success. Furthermore, the delivery of health information to people in a language that is not their first will be a specific contributary factor to their level of health literacy. This review identified only one study that sought to improve participants’ knowledge in a non-native language population [20]. Three other reviews conducted recently scrutinised the effect of animation only style interventions [29–31] and were also unable to identify robust data, other than the one publication also included in our review [26], that animations can increase knowledge amongst people who did not speak the country’s native language.

The content and duration of the MmET interventions reviewed were variable so it remains unclear which style is most effective, video (people) or animations, and whether any accompanying information should be textual or verbal, particularly for populations with a lower health literacy and who speak English (or any country’ native language) as an additional language. The relevance of the CTML [5] was acknowledged in only two papers in our review [14,26] and by the author of one other systematic review [29].

### Strengths and Limitations

This systematic review has several strengths: this was registered with PROSPERO, we worked with a professional librarian to develop the search strategy, and we followed the JBI methodology to structure the review and assess for risks of bias [11]. The search strategy included a wider definition of multimedia so incorporated publications reporting on video (people) interventions in addition to animations, in contrast to other recent systematic reviews [29–31]. However, this review is also subject to a number of limitations. Although the decision to focus on studies with a control group and to appraise the data quality may have enhanced the quality of the results, other helpful findings in evaluations or service improvement studies may have been missed. It was not possible to undertake a meta-analysis of results due to the differences in study designs (pilot studies, quasi- experimental and RCT), the content of and duration of the interventions, the different time-points when results were measured, and the variety of outcome measures used amongst the 15 publications in this review. The decision to restrict the inclusion of studies to three medical search engines and those published after 2014 means that otherwise eligible studies published before that date may have been overlooked.

### Future research

This review has identified several opportunities for future research. There is a need for data to confirm longer-term health knowledge retention, an increase engagement in clinical services, and improvements in clinical outcomes. Almost all of the research reviewed was conducted in high income countries, a feature also noted in other systematic reviews [29,30], so there is a need for data from low to middle income countries or with migrant populations from these locations. There is also a need for research to understand the format, duration, content, style, and efficacy of MmET interventions on populations with low levels of health literacy whose native language is not the same as that in which the intervention is delivered, to inform initiatives that can reduce health inequalities amongst global majority populations. Finally, research is required to understand if and how these interventions can be utilized by people who do not have digital literacy skills and access to the technology.

## Conclusions

In conclusion, the reviewed studies confirm that there is a positive directional link between multimedia interventions and improved levels of short-term knowledge in contrast to written material. The synthesized findings also identified several research gaps where there are opportunities to strengthen the understanding and application of MmET when delivering health information. This review highlights the positive impact of creative methods for delivering health knowledge but emphasizes too the need to consider the effects of health literacy, and integration of evidence when designing the content. The use of a MmET approach for health education is a beneficial approach for those from underserved communities, particularly when spoken or written English may be limited. This review provides evidence to support the development and use of MmET by community nurses during home visits, group health education sessions, or supporting self-care activities for people with long term conditions.

## Figures and Tables

**Figure I:**
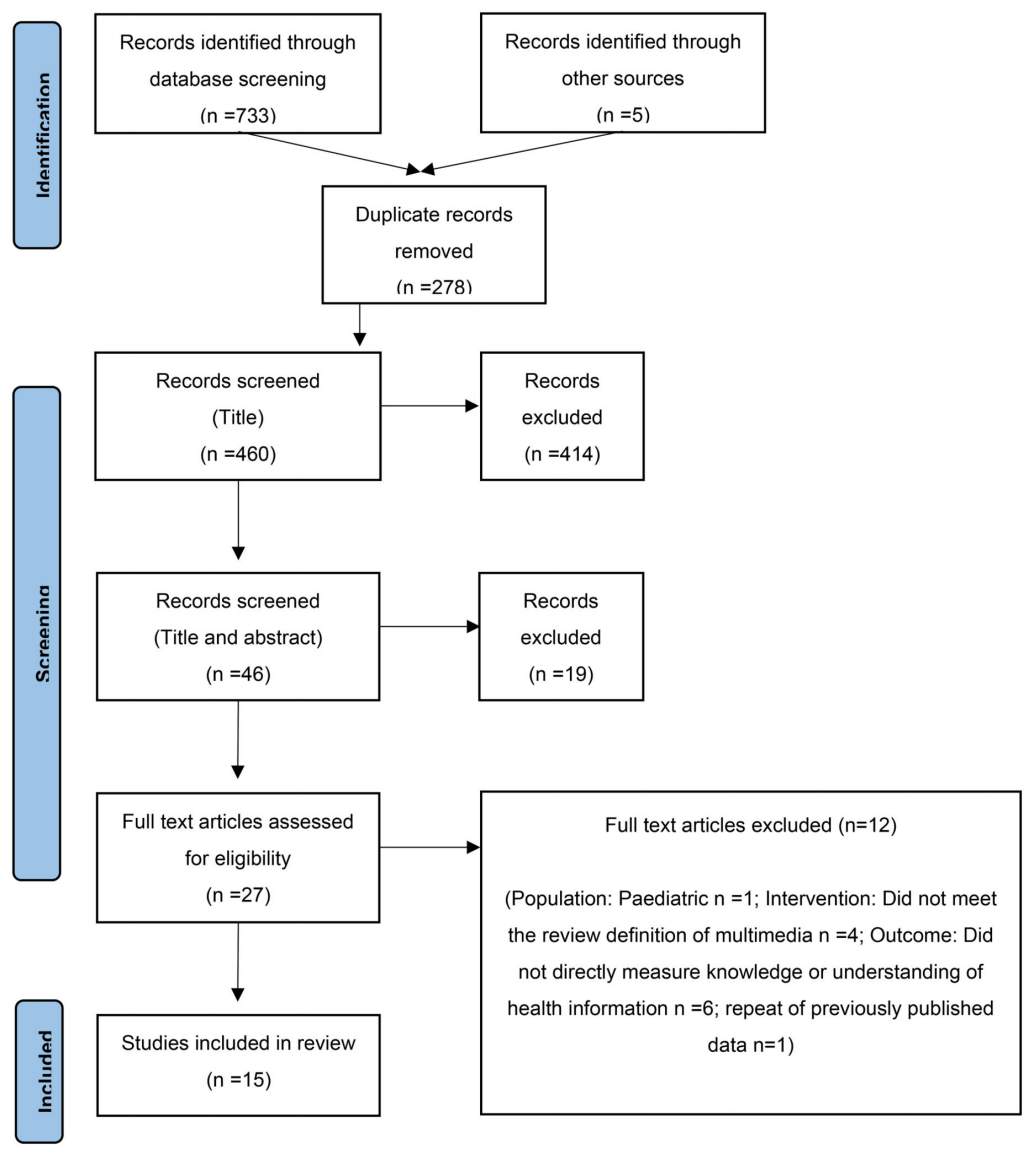
PRISMA flow diagram

**Table I:** (a) Overview of study characteristics

Study	Country	Population	Intervention type and duration	Education Topic/ Context	Results/Outcomes
Dan et al. 201 5	China	Rural Chinese people at risk of glaucoma or diabetic eye disease	Video (people) and spoken voice, not interactive, 10 mins	Comprehensive eye examination	Pre-video, most (74.7%) people could not answer one question. Post-video, mean scores increased by 1.39, p< 0.001.
Dathatri et al. 2014	US	First time elective cardiac catheterization	Animation and spoken voice, interactive elements, 20-40 mins	Percutaneous cardiac catheterization interventions	Both groups had more knowledge after their interventions, but no statistically significant differences between scripted verbal or written consent, or the video and web-based consent.
Dharmakulaseelan et al. 2019	Canada	People who have had a stroke or trans-ischaemic attack	Animation, written text and spoken voice, 5 mins	Continuous Positive Airways Pressure (CPAP)	Feasible and acceptable to deliver, but the intervention didn’t change knowledge or CPAP usage p= 0.352.
Ellett et al. 2014	Australia	Women with pelvic pain	Animation, written text and spoken voice, not interactive, 15 mins	Consent for a laparoscopy	At initial test, intervention group mean knowledge score of 11.3 vs control 7.9, p< 0.001. Six weeks later, no difference: intervention group 8.4 vs control 7.8, p= 0.44.
Ferguson et al. 2016	UK	First-time hearing aid users	7 X video (people), animations, spoken voice, written text, interactive, 60 mins	Using hearing aids	Six hypotheses tested, four confirmed: spoken messages better recall than written, animated with spoken is better than illustrations, especially in low literacy groups. All p< 0.5. Cohen’s effect size 0.94

(b) Overview of study characteristics					
Study	Country	Population	Intervention type and duration	Education Topic/ Context	Results/Outcomes
Meppelink et al. 2015	Netherlands	People aged over 55 and eligible for colorectal cancer screening	Animation or illustration, written text or spoken voice, no interactive elements, 15 web-pages, duration not stated	Colorectal cancer screening	High attrition between hearing aid fitting and evaluation so only those who completed are analysed. Increase in PHAST (practical hearing aid handling skills) and HACK (knowledge if hearing aids and communications) p< 0.001.
Moore et al. 2020	US	Patients requiring endoscopic siting of a pH monitoring capsule	Animation with spoken voice, no interactive elements, 4 mins	pH testing during an endoscopy	No difference in the proportion of people who stopped taking proton pump inhibitors pre procedure p= 0.85. Likely due to inadequate information in the animated video. People did improve diet and recording meals. Satisfaction scores throughout each stage improved with the video p< 0.05.
Parker et al. 2018	US	First time colonoscopy for bowel cancer screening	Animation, spoken voice and written text, interactive elements, duration not stated	Pre-endoscopy preparation	Increased knowledge in the intervention group 82% vs 74% correct p=< 0.005. 58% had reduced anxiety, 4% increased anxiety, 38% no difference, Intervention group required less sedation (midazolam 3.66mg vs 4.46mg p= 0.004) and a trend to less fentanyl. The procedure time in the intervention group was shorter 24.8 min vs 29.1 min p=0.02.

(c) Overview of study characteristics					
Study	Country	Population	Intervention type and duration	Education Topic/ Context	Results/Outcomes
Pei et al. 2017	China	Patients requiring an alginate oral impression	Video (people) and spoken voice, no interactive elements, 3 mins	Oral-impression taking pre-dental treatment	Control group mean knowledge = 29.84%, picture group 43.75%, and video group 53.85% p=<0.001. Patient satisfaction was highest in video, then picture, then control. good/very good 98.46%, 94.92%, 52.4%. Performance / compliance evaluation was highest in video, then picture, then control - 85.38%, 65.18%, 63.20%.
Peipert et al. 2021	US	Patients with stages 1-3 cancer receiving chemotherapy or radiation therapy	Video (people), animation, spoken voice and written text, interactive elements, duration not stated	Bowel and breast cancer	Health beliefs, self-efficacy, satisfaction of communication and HRQOL, all were not statistically significant and had a small Cohens effect size. Increase in cancer knowledge p= 0.5, Cohen’s effect size 0 .48.
Poureslami et al. 2016	Canada	Patients with COPD requiring culturally and linguistically specific education (Mandarin / Cantonese)	Video (people) and spoken voice, no interactive elements, two options either 12 or 20 mins	Chronic obstructive airways disease and inhaler technique	Change in inhaler technique: 3 intervention groups better than control, especially group 1 clinician video. All were p<0.001. Understanding pulmonary rehab: only groups 1 and 2 had a significantly better understanding than control p< 0.05. No benefits from seeing both videos.
Tait et al. 2014	US	Patients needing diagnostic cardiac catheterization	Animation, spoken voice and written text, interactive elements, 12 mins	Percutaneous cardiac catheterization interventions	The intervention group had improved knowledge recall post intervention and 2 weeks later compared to the usual care group. Post test 1: 8.28 vs 7.35, and post-test 2: 7.60 vs 6.69. p< 0.05.
Trinh et al. 2014	US	Kidney, liver, or lung transplant recipients	Video (people) with spoken voice, no interactive elements, 2 mins	Skin cancer	The video group had a greater post intervention score pre 3.96 vs the control group who had a pamphlet 1.76, p<0.001.

(d) Overview of study characteristics					
Study	Country	Population	Intervention type and duration	Education Topic/ Context	Results/Outcomes
Wierstra et al. 2018	Canada	Patients with IBD seeking reproductive health information	Animation, written text, interactive elements, 5 x 5 mins (25 mins total)	Reproductive health and irritable bowel disease	Overall pre/post-test knowledge median score for MM was 7 (pre) and 16 (post) and the text only median score was 8 (pre) and 15 (post). The median within-subject increase was 6.5, p=< 0.00. Overall MM knowledge gain vs text knowledge gain: 8 vs 6 p= 0.216.
Yap et al. 2020	Singapore	Patients requiring coronary angiography/plasty	Animation (whiteboard), spoken voice, no interactive elements, 3 mins	Percutaneous cardiac catheterization interventions	Intervention group: pre-7.60, post 10.25 p<0.001 but intervention scores pre 7.60 and control pre 8.49. Intervention group had lower baseline education levels (post A level) than the control group p=0.024.

**Table II:** JBI internal and external validity appraisal scores for each of the 15 publications reviewed.

VALIDITY:	INTERNAL										EXTERNAL			n= Score
Study	1	2	3	4	5	6	7	8	9	10	11	12	13	
Dan et al. (2015)	yes	unclear	yes	no	no	yes	yes	yes	yes	yes	yes	yes	yes	10
Dathatri et al. (2014)	yes	unclear	yes	no	no	yes	yes	yes	yes	yes	yes	yes	yes	10
Dharmakulaseelan et al. (2019)	yes	unclear	yes	no	no	yes	yes	yes	yes	yes	yes	yes	yes	11
Ellett et al. (2014)	yes	yes	yes	no	yes	yes	unclear	yes	yes	yes	yes	yes	yes	11
Ferguson et al. (2016)	yes	yes	yes	no	yes	yes	yes	yes	yes	yes	yes	yes	yes	11
Meppelink et al. (2015)	yes	yes	yes	no	yes	yes	yes	yes	yes	yes	yes	yes	yes	12
Moore et al. (2019)	yes	yes	yes	no	no	yes	yes	yes	yes	yes	yes	yes	yes	10
Parker et al. (2018)	yes	no	yes	yes	no	yes	unclear	yes	yes	yes	yes	yes	yes	10
Pei et al. (2017)	yes	yes	yes	no	yes	yes	yes	yes	yes	yes	yes	yes	yes	12
Piepert et al. (2021)	yes	yes	yes	no	no	yes	no	yes	yes	yes	yes	yes	yes	10
Poureslami et al. (2016)	yes	yes	yes	no	yes	yes	yes	yes	yes	yes	yes	yes	yes	12
Tait et al. (2014)	yes	unclear	yes	no	yes	yes	yes	yes	yes	yes	yes	yes	yes	10
Trinh et al. (2014)	yes	no	yes	no	no	yes	no	yes	yes	yes	yes	yes	yes	9
Wierstra et al. (2018)	unclear	no	yes	no	no	n/a	n/a	yes	yes	yes	yes	yes	yes	9
Yap et al. (2019)	yes	yes	yes	no	no	yes	unclear	yes	yes	yes	yes	yes	yes	10

## Data Availability

Data sharing is not applicable to this systematic review article as no new data were created or analyzed in this study.

## Box I Search terms used during electronic literature searching of papers for inclusion.

1. ((audiovisual or multimedia) and health and “patient education”).mp. [mp=ti, ab, hw, tn, ot, dm, mf, dv, kf, fx, dq, bt, nm, ox, px, rx, an, ui, sy, ux, mx] 1,782
2. limit 1 to english 1,717
3. limit 2 to yr=“2014 -Current” 751
4. remove duplicates from 3 485
5. 4 and “Randomized Controlled Trial”.sa_pubt. 43
